# HBP1 inhibits the development of type 2 diabetes mellitus through transcriptional activation of the *IGFBP1* gene

**DOI:** 10.18632/aging.204364

**Published:** 2022-11-02

**Authors:** Yuning Cheng, Ruixiang Yang, Yue Zhou, Jiyin Wang, Tongjia Zhang, Shujie Wang, Hui Li, Wei Jiang, Xiaowei Zhang

**Affiliations:** 1 Department of Biochemistry and Biophysics, School of Basic Medical Sciences, Beijing Key Laboratory of Protein Posttranslational Modifications and Cell Function, Peking University Health Science Center, Beijing 100191, P. R. China

**Keywords:** HBP1, IGFBP1, T2DM, insulin resistance, glucose metabolism

## Abstract

Type 2 diabetes mellitus (T2DM) is a chronic metabolic disease that is highly prevalent worldwide and characterized by glucose and lipid metabolism disorders. However, the pathogenic mechanisms have not been fully established. Here, we found that HMG-box transcription factor 1 (HBP1) is involved in T2DM and that its deficiency in mice aggravates the features of diabetes. In addition, we undertook screening by RNA sequencing and found that HBP1 activates the transcription of the insulin-like growth factor binding protein 1 (*IGFBP1*) gene. Moreover, Insulin and palmitic acid reduced HBP1 protein expression and inhibited its binding to the *IGFBP1* promoter. Furthermore, HBP1 reduced the serum free insulin-like growth factor 1 (IGF-1) concentration through IGFBP1 and inhibited the PI3K/AKT signaling pathway. This forms an insulin/HBP1/IGFBP1 negative feedback regulatory loop to dynamically regulate blood glucose and insulin concentrations. These findings have elucidated a mechanism whereby HBP1 and its negative feedback regulatory loop influence the development of T2DM, thereby providing a new theoretical basis and potential therapeutic target for T2DM.

## INTRODUCTION

Type 2 diabetes mellitus (T2DM) is a chronic and metabolic disease that is caused by a variety of factors [1]. The common manifestations of T2DM are hyperglycemia, insulin resistance, impaired or compensatory insulin secretion, pancreatic β-cells hyperplasia, and abnormal carbohydrate, fat, and protein metabolism [1–3]. T2DM is often accompanied by nonalcoholic fatty liver disease (NAFLD), which is also characteristic of the metabolic syndrome [1, 4]. Numerous studies have shown that high-fat diets (HFDs) are a principal cause of NAFLD, and induce chronic liver inflammation and insulin resistance, which can lead to T2DM [1, 5]. During the last few decades, the prevalence of T2DM has risen rapidly around the world [1, 6], and T2DM itself and the associated severe complications represent frequent causes of death and disability worldwide. Therefore, there is an urgent need to explore the pathogenetic mechanism of these diseases to identify therapeutic targets for T2DM.

High mobility group (HMG) box-containing protein 1 (HBP1) is a ubiquitously expressed transcription factor that belongs to the sequence-specific HMG family. Previous studies have shown that HBP1 has dual transcriptional effects. It primarily acts as a transcriptional repressor, targeting the N-Myc (*MYCN*), p47phox (*NCF1*), AFP, DNA-methyltransferase 1 (*DNMT1*), *EZH2*, and *MIF* genes by directly binding to the high-affinity response elements on their promoters [7–12]. Moreover, HBP1 can also activate the transcription of specific genes, such as *p16INK4A*, *p21CIP1*, histone H1, *CD2* and myeloperoxidase (*MPO*) [13–17]. HBP1 plays a vital role in cell differentiation and cell cycle progression by regulating the expression of such target genes. The overexpression of HBP1 arrests cell cycle and inhibits tumorigenesis, which implies that HBP1 functions as a tumor suppressor. It has been reported that HBP1 can inhibit Srebp1c through p53, thus inhibiting lipid synthesis [18]. Owing to the close relationships between glucose metabolism and lipid metabolism, we hypothesized that HBP1 may also play a role in glucose metabolism and be involved in T2DM. However, whether HBP1 is involved in glucose metabolism or T2DM and the mechanisms involved remain unclear.

Although insulin plays a fundamental role in maintaining normal blood glucose concentration, it is widely believed that the related peptides, the insulin-like growth factors (IGFs), and especially IGF-1, play complementary roles in glucose regulation [19, 20]. IGF-I, a peptide hormone, shares amino acid sequence homology with insulin and has insulin-like activity. IGF-1 can reduce blood glucose by promoting glucose uptake by peripheral tissues, inhibiting glucose production or promoting glycogen synthesis by the liver. Both insulin and IGF-1 reduce blood glucose through activation of PI3K/AKT signaling pathway [21–24]. IGF binding proteins (IGFBPs) are key regulators of the IGF axis, and therefore may also affect the regulation of glucose metabolism [19, 25, 26].

IGFBP1 is a member of the IGFBP family, is mainly expressed in the liver, and circulates in the plasma [25, 27]. IGFBP1 can bind IGF-1, thereby prolonging its half-life and competitively inhibiting its interaction with its cell surface receptors [20, 28]. Therefore, IGFBP1 can have effects on metabolism by affecting the level of activation of the IGF-1/PI3K/AKT axis. Low concentrations of IGFBP1 may be associated with impaired glucose tolerance in human patients [29, 30]. Furthermore, IGFBP1 is regulated by hormones, such as insulin: insulin reduces the expression of *IGFBP1* by the insulin response element (IRE) on its promoter [31–34].

In the present study, we aimed to characterize the role of HBP1 in T2DM through its regulation of the *IGFBP1* gene, and found that the insulin/HBP1/IGFBP1/IGF-1/PI3K/AKT axis is critical for blood glucose regulation, because the deletion of HBP1 disrupts glucose homeostasis and induces T2DM.

## RESULTS

### Hepatic HBP1 expression is low in mouse models of T2DM

**Figure 1:**
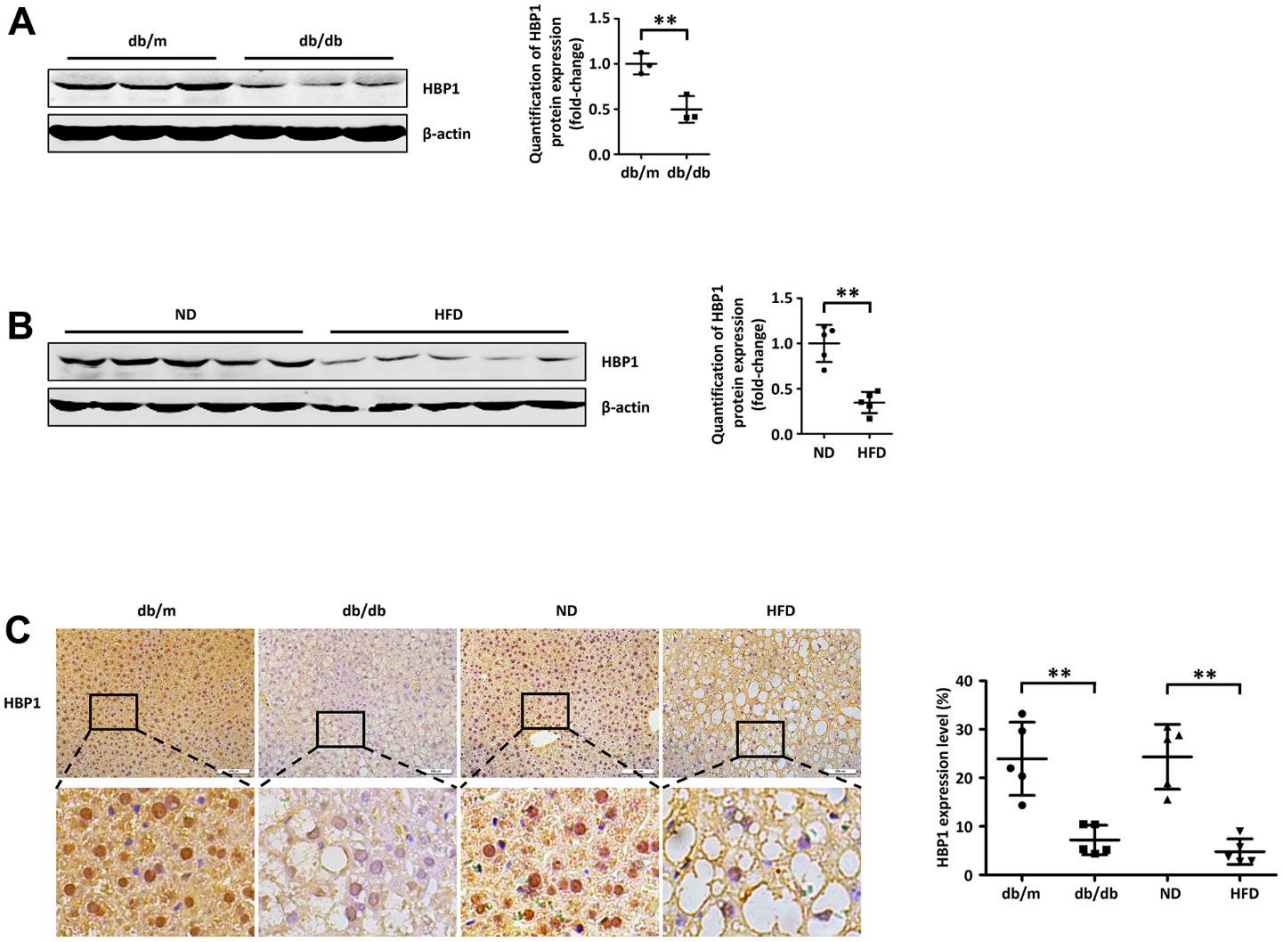
**Hepatic HBP1 expression is low in mouse models of T2DM.** (**A**) HBP1 protein level reduces in adult db/db mice. The protein of endogenous HBP1 was extracted from db/m and db/db mice (n=3) livers and was measured by western blotting. β-actin was detected as a loading control. Quantification was normalized to β-actin. (**B**) HBP1 protein level reduces in C57BL/6J mice fed by high fat diet. The protein of endogenous HBP1 was extracted from livers (n=5) of wild type (WT) C57BL/6J mice fed by normal diet (ND) and high fat diet (HFD), and was measured by western blotting. β-actin was detected as a loading control. Quantification was normalized to β-actin. (**C**) The HBP1 content in livers of type 2 diabetic model mice is low. Liver sections collected from db/m, db/db, ND and HFD mice (n=5) were immunohistochemically stained with anti-HBP1. Scale bar, 100 μm. Stained areas were quantitated by Image J software. Error bars represent S.D. **, p<0.01.

To characterize the relationship of the transcription factor HBP1 with T2DM, we measured HBP1 protein expression in the livers of *db*/*db* mice and HFD-fed mice, both of which develop diabetes. First, we collected livers from adult *db*/*db* mice and *db*/m controls, and found that the protein expression of HBP1 was lower in the *db*/*db* mice (Figure 1A). Next, we induced diabetes in C57BL/6J mice by feeding them an HFD for 3 months, thus creating a classical mouse model of T2DM, then collected livers from C57BL/6J mice fed the HFD or a normal diet for the measurement of HBP1 protein content by western blotting. As shown in Figure 1B, the liver HBP1 protein expression of mice fed the HFD was lower than that of mice fed the normal diet. Furthermore, we prepared sections of the livers of each group of mice for immunohistochemical analysis. Consistent with the findings of a previous study, HBP1 was principally localized to the nucleus [35]. In addition, there was significantly lower HBP1 staining in both *db*/*db* and HFD-fed mice with T2DM, compared with the equivalent control mice (Figure 1C). To determine whether HBP1 protein expression was also associated with T1DM, we constructed of T1DM mice model using streptozotocin induction. Then we tested HBP1 protein levels in liver of T1DM mice. As shown in (Supplementary Figure 1), HBP1 protein levels were unchanged in T1DM mice compared with wild-type mice, indicating that the regulatory mechanism of HBP1 has no effect on the occurrence of T1DM. Thus, HBP1 protein expression is lower in the livers of mice with T2DM and may be involved in the development of T2DM.

### HBP1 knockout mice show a significant worsening of diabetes

**Figure 2:**
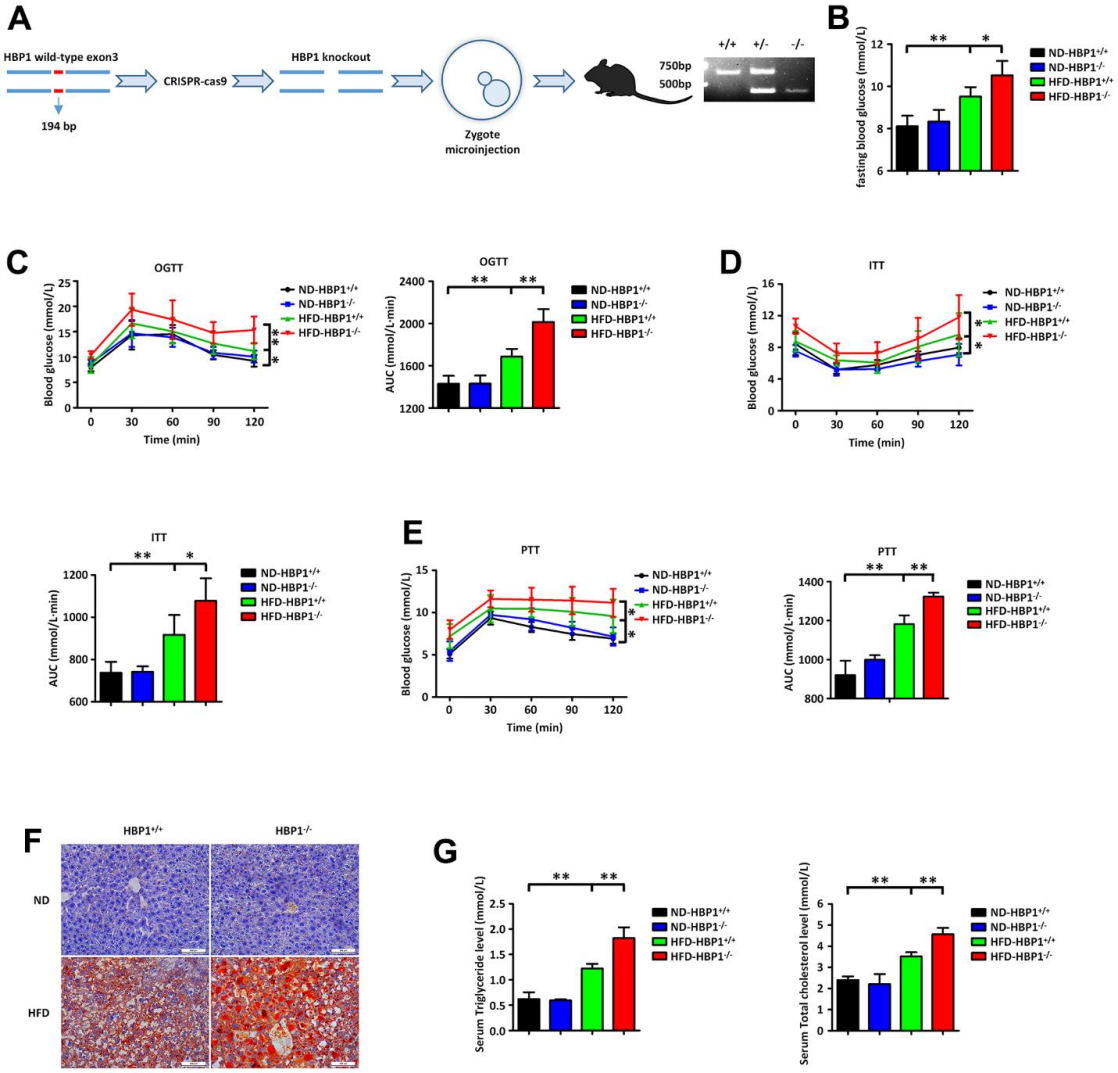
***HBP1* knockout mice show a significant worsening of diabetes.** (**A**) Schematic of the *HBP1* knockout (HBP1^−/−^) mice and identification of genotypes. The genotypes were identified by PCR. The PCR product of wild type mice has only one band at the position of 611bp. The PCR product of heterozygous mice has one band at the position of 611bp and another one at the position of 417bp. The PCR product of HBP1^−/−^ mice has only one band at the position of 417bp. (**B**) HBP1^−/−^ mice fed by HFD has higher fasting blood glucose. Wild type C57BL/6J (HBP1^+/+^) mice and HBP1^−/−^ mice of the same age were both divided into two groups randomly. One group was fed by ND as a control and the other was fed by HFD for 3 months to induce to diabetic mice. These four groups were fasted for 6 hours and drank water freely. After that, blood was collected from the tail vein of mice and its glucose content was measured by Roche glucometer. (**C**) HFD-HBP1^−/−^ mice have more severe impaired glucose tolerance. All the four groups were fasted for 6 hours and drank water freely. After 6 hours, fasting blood glucose was recorded as 0 min point. Each mouse was given 5 mg/kg glucose by gavage and its glucose content in tail vein blood was measured by Roche glucometer every 30 minutes, up to 2 hours. (**D**) HFD-HBP1^−/−^ mice are less sensitive to insulin. All the four groups were fasted for 4 hours and drank water freely. After 4 hours, fasting blood glucose was recorded as 0 min point. Each mouse was given 1 U/kg insulin by intraperitoneal injection and its glucose content in tail vein blood was measured by Roche glucometer every 30 minutes, up to 2 hours. (**E**) HFD-HBP1^−/−^ mice have higher glucose excursion during pyruvate tolerance test. All the four groups were fasted for 16 hours and drank water freely. After 16 hours, fasting blood glucose was recorded as 0 min point. Each mouse was given 1 g/kg pyruvate by intraperitoneal injection and its glucose content in tail vein blood was measured by Roche glucometer every 30 minutes, up to 2 hours. (**F**) The livers of HFD-HBP1^−/−^ mice have more severe steatosis. Representative photographs of liver samples were selected and analyzed by oil red O staining. Scale bar, 100 μm. (**G**) Serum triglyceride and total cholesterol contents in HFD-HBP1^−/−^ mice are significantly increased. Serums were isolated from the inferior vena cava. AUC, Area Under Curve. Data were the mean ± SD by a one-way ANOVA. *, p<0.05. **, p<0.01.

To explore the role of HBP1 in T2DM, we constructed guided RNA targeting the *HBP1* gene and used CRISPR-Cas9 to knock out this gene (Figure 2A, left panel). After obtaining founder C57BL/6J mice by zygote microinjection, we established an HBP1 knockout strain (HBP1^−/−^) through mating with wild-type mice and identifying mice with the appropriate genotype by PCR (Figure 2A, right panel). Next, we randomly allocated the HBP1^−/−^ mice to two groups, one of which was fed the normal diet (ND-HBP1^−/−^), while the other was fed the HFD, for 3 months to induce diabetes (HFD-HBP1^−/−^). In addition, wild-type C57BL/6J mice were similarly grouped and fed, creating ND-HBP1^+/+^ and HFD-HBP1^+/+^ groups. We then measured the fasting blood glucose concentrations of the mice in blood samples obtained from tail veins using a glucometer. As shown in Figure 2B, the fasting blood glucose concentrations of the HFD-HBP1^−/−^ and HFD-HBP1^+/+^ mice were significantly higher than those of the ND-HBP1^−/−^ and ND-HBP1^+/+^ mice. In addition, the HFD-HBP1^−/−^ mice had higher fasting blood glucose concentrations than the HFD-HBP1^+/+^ mice, but there was no difference between the concentrations of the ND-HBP1^−/−^ and ND-HBP1^+/+^ mice. Thus, HFD-feeding increases the fasting blood glucose concentrations of both HBP1^−/−^ and HBP1^+/+^ mice, but the effect was more marked in the HBP1^−/−^ mice.

In addition, we performed oral glucose tolerance testing (OGTT), insulin tolerance testing (ITT), and pyruvate tolerance testing (PTT) in the four groups of mice. Consistent with the fasting blood glucose data, HFD-HBP1^−/−^ showed the highest glucose excursions during OGTT (Figure 2C) and PTT (Figure 2E) and the lowest sensitivity to insulin during ITT (Figure 2D); and HFD-HBP1^+/+^mice showed more modest differences from the control groups. There were no differences between the ND-HBP1^−/−^ and ND-HBP1^+/+^ groups. These findings demonstrate that the knockout of the *HBP1* gene further worsens the glucose homeostasis of mice with HFD-induced diabetes, whereas it does not alter the glucose homeostasis of mice consuming a normal diet.

Because T2DM is often accompanied by NAFLD, we collected livers from the four groups of mice and stained them with oil red O to evaluate lipid deposition (Figure 2F). This showed that the HFD-HBP1^−/−^ mice also had more severe lipid deposition in their livers. We also collected blood samples from the caudal venae of the mice and measured their serum triglyceride and cholesterol concentrations. As shown in Figure 2G, the serum triglyceride and cholesterol concentrations of the HFD-HBP1^−/−^ mice were higher than those of the HFD-HBP1^+/+^ mice. These results imply that HBP1 knockout alone does not affect overall glycolipid homeostasis in mice fed a normal diet. However, when challenged with an HFD, the consumption of which is a key risk factor for T2DM [36], HBP1 knockout worsened the diabetes and fatty liver of the mice.

### HBP1 increases the expression of IGFBP1 through a transcriptional effect

**Figure 3:**
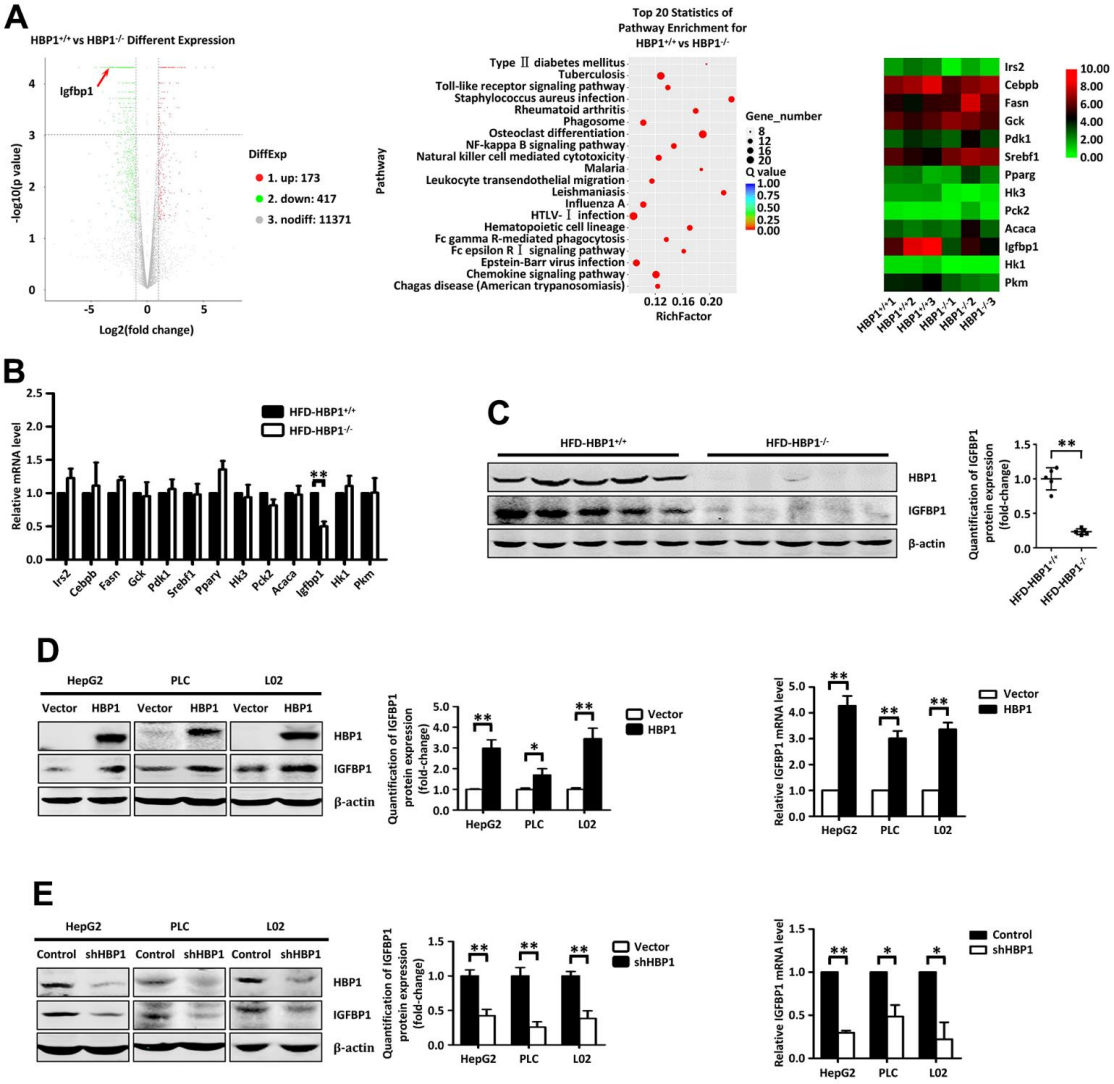
**HBP1 increases the expression of IGFBP1 by binding to the *IGFBP1* promoter.** (**A**) RNA sequencing analysis of liver tissues from HBP1^+/+^ and HBP1^−/−^ mice. (**B**) HBP1 affects the mRNA level of IGFBP1 in mouse liver tissues. The mRNA levels of Irs2, Cebpb, Fasn, Gck, Pdk1, Srebf1, Pparγ, Hk3, Pck2, Acaca, Igfbp1, Hk1 and Pkm2 were measured by real-time PCR in liver tissues from HBP1^+/+^ and HBP1^−/−^ mice. (**C**) HBP1 affects the protein level of IGFBP1 in mouse liver tissues. The protein levels of HBP1 and IGFBP1 were measured by western blotting in liver tissues from HBP1^+/+^ and HBP1^−/−^ mice. β-actin was used as a loading control. Quantification was normalized to β-actin, n = 5 mice per group. (**D**) HBP1 overexpression promotes both protein and mRNA expression of IGFBP1 in HepG2, PLC/PRF/5 and L02 cells. The cells were transfected with HA-HBP1 or pcDNA3 (as a control). The protein levels of HBP1 and IGFBP1 were measured by western blotting (left panel). β-actin was used as a loading control. Quantification was normalized to β-actin. The mRNA level of IGFBP1 was measured by real-time PCR (right panel). (**E**) *HBP1* knockdown inhibits both protein and mRNA expression of IGFBP1 in HepG2, PLC/PRF/5 and L02 cells. The cells were stably transfected with pLL3.7-shHBP1 or pLL3.7 (as a control) through lentiviral infection. The protein levels of HBP1 and IGFBP1 were measured by western blotting (left panel). β-actin was used as a loading control. Quantification was normalized to β-actin. The mRNA level of IGFBP1 was measured by real-time PCR (right panel). Data were the mean ± SD by a two-tail, unpaired Student’s t-test. *, p<0.05. **, p<0.01.

To investigate the mechanism whereby HBP1 may predispose toward T2DM, we performed RNA sequencing of livers from ND-HBP1^−/−^ and ND- HBP1^+/+^ mice and constructed a volcano plot (Figure 3A, left panel). This shows 590 differentially expressed genes, of which 173 were upregulated and 417 were downregulated in ND-HBP1^−/−^ mice *versus* ND-HBP1^+/+^ mice. Kyoto Encyclopedia of Genes and Genomes (KEGG) pathway enrichment analysis showed that some of the enriched genes are members of pathways related to T2DM (Figure 3A, middle panel). A heatmap of the 13 most significantly differentially expressed genes involved in glucolipid metabolism is shown in Figure 3A (right panel). To further determine which genes were affected by HFD-feeding, we extracted RNA from the livers of HFD-HBP1^−/−^ mice and control HFD-HBP1^+/+^ mice and performed real-time PCR assays. As shown in Figure 3B, only the IGFBP1 mRNA expression was lower in the livers of HFD-HBP1^−/−^ mice than in those of HFD-HBP1^+/+^ mice, whereas the mRNA expression of the other 12 genes did not significantly differ. In addition, we performed western blotting for IGFBP1 protein, and confirmed that this was also lower in the HFD-HBP^−/−^ mice (Figure 3C). These findings imply that *IGFBP1* may be a target gene of HBP1 in mice and that the regulation of *IGFBP1* transcription by HBP1 may account for the more severe T2DM in HFD-HBP1^−/−^ mice.

We then wondered if this regulation of IGFBP1 by HBP1 would also occur in human cells. Therefore, we exogenously overexpressed HBP1 in human hepatocellular carcinoma-derived HepG2 cells, PLC hepatoma cells, and normal liver L02 cells, and this increased both the protein and mRNA expression of IGFBP1 in all three cell lines (Figure 3D). To further test our hypothesis, we used lentiviral vectors encoding short hairpin RNAs (shRNAs) to knock down HBP1 expression in the three cell lines, which reduced the expression of IGFBP1 at both the protein and mRNA levels (Figure 3E). In addition, we also detected the mRNA and protein levels of IGFBP3, which is highly homologous to IGFBP1, in HepG2, PLC or L02 cells with HBP1 overexpression or knockdown. As shown in (Supplementary Figure 2), the mRNA and protein levels of IGFBP1 were unchanged, indicating that HBP1 had no transcriptional regulation effect on IGFBP3. These data confirm that HBP1 increases the expression of IGFBP1 in mice and human liver cells through a transcriptional effect.

### HBP1 increases the expression of IGFBP1 by binding to the IGFBP1 promoter

**Figure 4:**
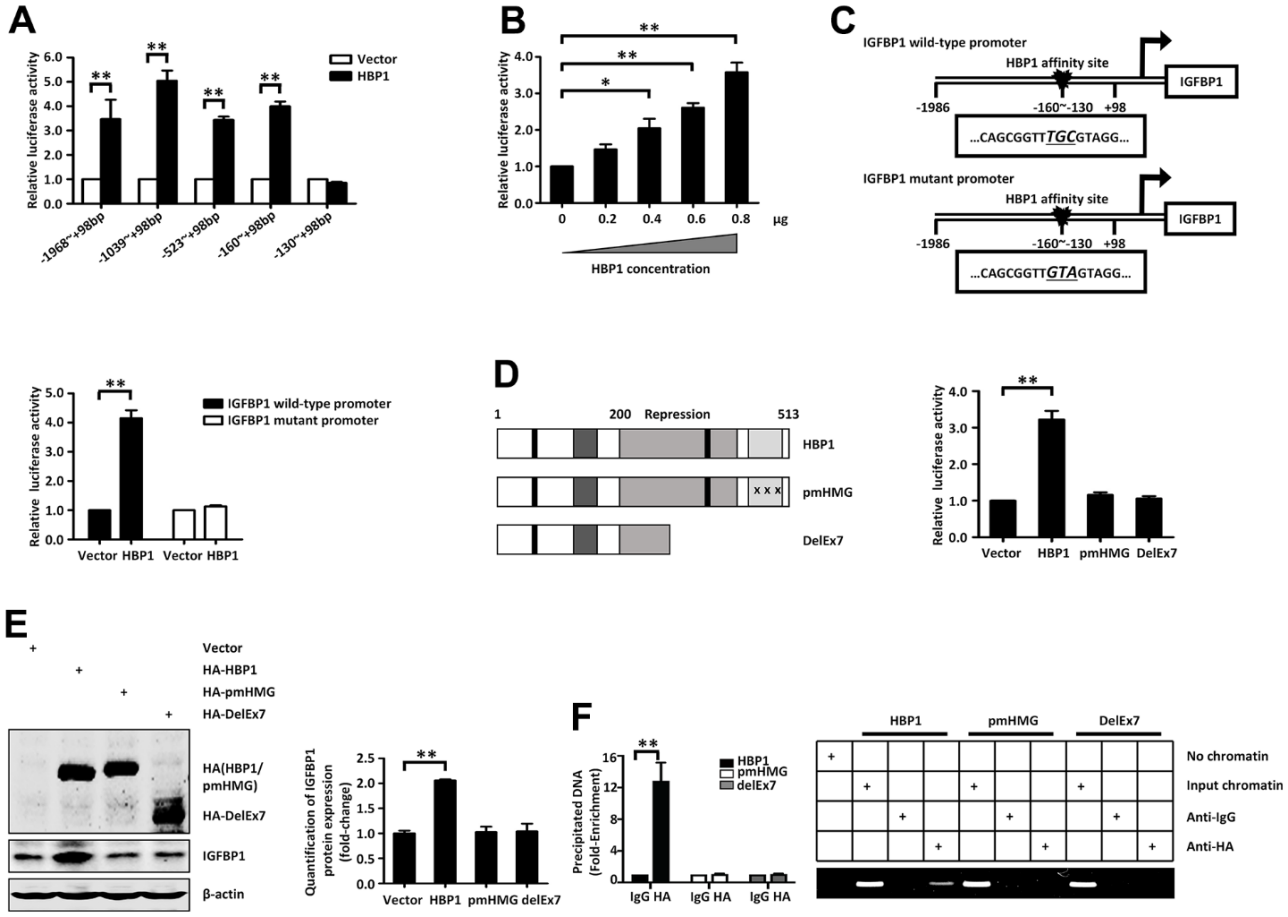
**Insulin and palmitic acid reduce the protein expression of HBP1 and the binding of HBP1 to the *IGFBP1* promoter.** (**A**) The relative luciferase activities of HBP1 on *IGFBP1* promoters of different lengths. 293T cells were co-transfected with the indicated length of *IGFBP1* promoter and HA-HBP1 plasmid. Luciferase activities were determined 24 hours after transfection and were analyzed from four separate experiments. (**B**) HBP1 enhances *IGFBP1* promoter activity in a dose-dependent manner. 293T cells were co-transfected with full-length *IGFBP1* promoter and different doses (0, 0.2, 0.4, 0.6, 0.8 μg) of HA-HBP1 plasmid. The luciferase activities were analyzed as the means ± S.D. from four separate experiments. (**C**) The integrity of affinity sites is indispensable for HBP1 up-regulating *IGFBP1* promoter *in vivo*. The schematic diagrams of the wild-type *IGFBP1* promoter and its mutant promoter are shown in top panel. The mutant promoter of *IGFBP1* contains four point mutations at the HBP1 affinity site (TCAA to CTGG) in comparison with wild-type *IGFBP1* promoter. The relative luciferase activities of HBP1 on wild-type and mutant *IGFBP1* promoter were analyzed from four separate experiments and shown in bottom panel. (**D**) Wild-type HBP1 rather than its mutants activates *IGFBP1* promoter activities. Schematic diagrams of wild-type HBP1 and its associated mutants are shown in left panel. 293T cells were co-transfected with wild type *IGFBP1* promoter and HBP1 or its mutant plasmids. The luciferase activities were analyzed from four separate experiments (right panel). (**E**) Expression of exogenous HBP1 increases IGFBP1 protein level. 293T cells were transfected with HBP1 and its associated mutants. The protein level of HA (HA-HBP1, HA-pmHMG and HA-delEx7) and IGFBP1 was measured by Western blotting. β-actin was used as a loading control. Quantification was normalized to β-actin. (**F**) The combination of HBP1 and endogenous *IGFBP1* promoter depends on the HMG domain of HBP1. ChIP assays were performed to verify the binding of exogenous HBP1 to the endogenous *IGFBP1*. 293T cells were transfected with HA-HBP1 or HA-pmHMG or HA-DelEx7. The region from position -224 to -6 contains the HBP1 affinity site and was analyzed by specific PCR (right panel) and Realtime-PCR (left panel). Anti-HA antibody was used in the indicated lanes. Data were the mean ± SD by a two-tail, unpaired Student’s t-test. *, p<0.05. **, p<0.01.

Next, we planned to investigate whether the regulation of the activity of the IGFBP1 promoter by HBP1 requires DNA binding. We constructed a full-length promoter-containing plasmid (from −1,986 bp to +98 bp), according to the transcription start site, and a series of truncated segments as reporter genes. We co-transfected 293T cells with an HBP1 expression plasmid and reporter plasmids containing various lengths of the IGFBP1 promoter. As shown in (Figure 4A), HBP1 activated the luciferase reporter fused to the IGFBP1 promoter sequences, as long as they contained the sequence from −160 bp to −130 bp, whereas it had no effect on the −130 bp to +98 bp sequence. Thus, the HBP1 affinity site on the IGFBP1 promoter is located between −160 bp and −130 bp. In addition, HBP1 increased IGFBP1 promoter activity in a dose-dependent manner (Figure 4B).

We next studied the characteristics of the HBP1 affinity site described in a previous study [35], and identified a probable binding site (GTTTGCG) within this region. To confirm that this sequence is the DNA-binding region that is necessary for the regulation of gene expression by HBP1, we constructed a mutant reporter plasmid for the *IGFBP1* promoter with a point mutant at −142 bp (changing TGC to GTA) (Figure 4C, top panel). We found that HBP1 activated the luciferase of the wild-type *IGFBP1* promoter, but had no effect on the mutated version (Figure 4C, bottom panel), indicating that the integrity of this site is required for transactivation by HBP1.

To further investigate whether the transcriptional activation of *IGFBP1* by HBP1 is dependent on the DNA-binding domain of HBP1, we utilized two kinds of HBP1 mutants [8]: pmHMG, which has three amino acid mutations in the HMG domain that eliminate its DNA-binding ability, and delEx7, which was isolated from a breast cancer and lacks the entire DNA-binding domain and part of the repression domain (Figure 4D, left panel). Luciferase assay and western blotting showed that only the overexpression of wild-type HBP1 increased the luciferase activity of the IGFBP1 promoter (Figure 4D, right panel) and the protein expression of IGFBP1 (Figure 4E), respectively. In contrast, the overexpression of either pmHMG or delEx7 had little impact. Subsequently, we transfected HEK293T cells with HBP1, pmHMG, or delEx7 plasmids and performed chromatin immunoprecipitation (ChIP) and ChIP-qPCR to determine whether HBP1 directly binds to the *IGFBP1* promoter. Figure 4F showed that HBP1, but not pmHMG or delEx7, directly bound to the specific response element of the *IGFBP1* promoter, indicating that the DNA-binding domain is also essential for the transcriptional activation of the *IGFBP1* promoter by HBP1. In addition, we repeated luciferase and ChIP experiments in HepG2 cells Supplementary Figure 3, and the results were consistent with those in HEK293T cells. HBP1 activated IGFBP1 promoter activity and directly bound to IGFBP1 promoter in HepG2 cells. Taken together, the present findings show that HBP1 increases the expression of IGFBP1 through direct binding to a specific affinity site in the *IGFBP1* promoter.

### Insulin and palmitic acid reduce the protein expression of HBP1 and the binding of HBP1 to the IGFBP1 promoter

Insulin and palmitic acid (PA) both affect glucose and lipid metabolism [3, 37]. Given that the above findings suggest that HBP1 is involved in glucolipid metabolism and T2DM, we wondered whether these substances would affect HBP1 gene expression and/or its transcriptional regulatory activity.

**Figure 5:**
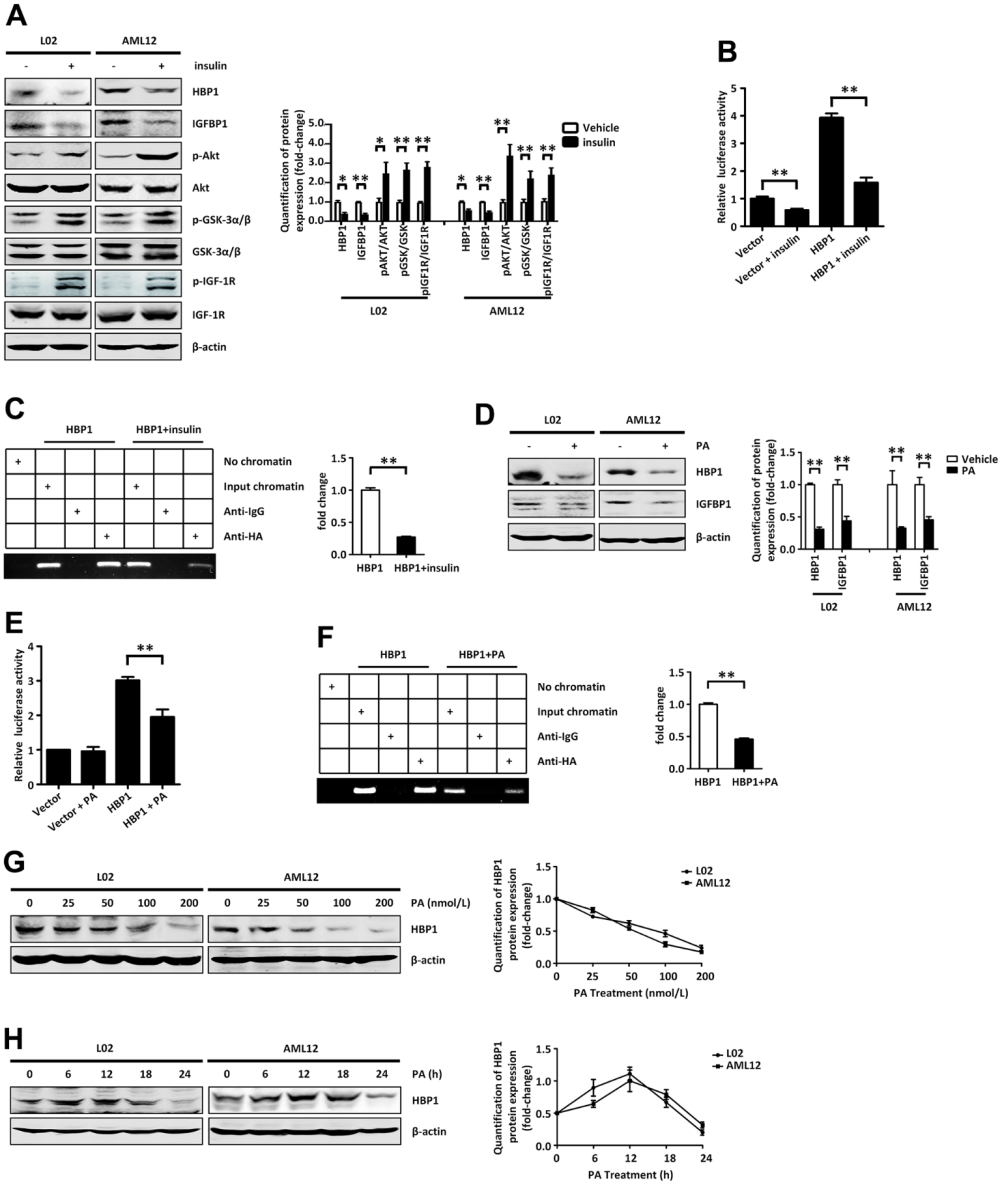
**Insulin and palmitic acid suppress the protein expression of HBP1 and the combination of HBP1 and *IGFBP1* promoter.** (**A**) Insulin activates p-AKT signaling pathway and inhibits protein expression of HBP1 and IGFBP1. The protein levels of HBP1, IGFBP1, p-AKT, p-GSK-3α/β, p-IGF-1R, AKT, GSK-3α/β and IGF-1R were measured by western blotting in L02 and AML12 cells. β-actin was used as a loading control. Quantification was normalized to β-actin. (**B**) Insulin can attenuate both the *IGFBP1* promoter activity and the activation effect of HBP1 on *IGFBP1* promoter. 293T cells were co-transfected with the *IGFBP1* promoter and HA-HBP1 plasmid and were treated with or without insulin. Luciferase activities were determined 24 hours after transfection and were analyzed from four separate experiments. (**C**) Insulin inhibits the binding ability of HBP1 and *IGFBP1* promoter. ChIP assays were carried out to verify the binding of exogenous HBP1 to the endogenous *IGFBP1*. 293T cells transfected with HA-HBP1 were treated with or without insulin. The region of *IGFBP1* promoter contains the HBP1 affinity site and was analyzed by specific PCR. Anti-HA antibody was used in the indicated lanes. Lanes were quantitated by Image J software. (**D**) Palmitic acid inhibits protein expression of HBP1 and IGFBP1. The protein levels of HBP1 and IGFBP1 were measured by western blotting in L02 and AML12 cells. β-actin was used as a loading control. Quantification of HBP1 and IGFBP1 protein expression was normalized to β-actin. (**E**) Palmitic acid weaken the luciferase activities of HBP1 on *IGFBP1* promoter. 293T cells were co-transfected with the *IGFBP1* promoter and HA-HBP1 plasmid and were treated with or without palmitic acid. Luciferase activity was determined 24 hours after transfection and analyzed from four separate experiments. (**F**) Palmitic acid restrains the binding ability of HBP1 and *IGFBP1* promoter. ChIP assays were performed to verify the binding of exogenous HBP1 to the endogenous *IGFBP1* gene. 293T cells transfected with HA-HBP1 were treated with or without palmitic acid. The region of *IGFBP1* promoter contains the HBP1 affinity site and was analyzed by specific PCR. Anti-HA antibody was used in the indicated lanes. Lanes were quantitated by Image J software. (**G**) The protein level of HBP1 decreases gradually with the increasing of PA dose. The protein levels of HBP1 were measured by western blotting in L02 and AML12 cells. β-actin was used as a loading control. Quantification was normalized to β-actin. (**H**) With the increase of PA duration, the protein level of HBP1 firstly increased and then decreased significantly. The protein levels of HBP1 were measured by western blotting in L02 and AML12 cells. β-actin was used as a loading control. Quantification was normalized to β-actin. Data were the mean ± SD by a two-tail, unpaired Student’s t-test. *, p<0.05. **, p<0.01.

Treatment of normal human hepatocytes (L02 cells) and normal mouse hepatocytes (AML12 cells) with insulin activated the insulin signaling pathway (Figure 5A) and reduced the protein levels of HBP1 and IGFBP1. It has previously been shown that IGFBP1 expression is regulated by hormones in humans, and insulin in particular plays a leading role in the regulation of IGFBP1 [28]. It inhibits the expression of IGFBP1 at the transcriptional level through IRE on the IGFBP1 promoter [32–34]. Therefore, we wondered whether insulin might affect the transcriptional activation of *IGFBP1* by HBP1. We co-transfected 293T cells with an *IGFBP1* promoter plasmid, containing the HBP1 affinity site, and the HBP1 expression plasmid or control plasmid. Twenty-four hours following transfection, we treated the cells with or without insulin, and found that insulin not only inhibited the luciferase activity via the IGFBP1 promoter, but also reduced the HBP1-induced IGFBP1 promoter activity (Figure 5B). We next transfected 293T cells with the HBP1 expression plasmid, treated them with or without insulin, and performed a ChIP assay. As shown in (Figure 5C), insulin reduced the binding of HBP1 to the *IGFBP1* promoter, which may explain the impaired HBP1-associated transcriptional regulation.

As shown in Figure 1, we found that HBP1 protein was expressed at lower levels in the livers of HFD-fed mice. This led us to wonder if PA, a saturated fatty acid found in almost all fats [37], would also alter HBP1 protein expression and affect the transcriptional regulation of the *IGFBP1* promoter by HBP1. We treated L02 and AML12 cells with PA for 24 hours and found that this reduced the protein levels of HBP1 and IGFBP1 by western blotting (Figure 5D), as for insulin treatment. Next, we co-transfected 293T cells with a full-length *IGFBP1* promoter plasmid and the HBP1 expression plasmid or the control plasmid and treated them with or without PA for 24 hours. Next, we co-transfected 293T cells with a full-length *IGFBP1* promoter plasmid and the HBP1 expression plasmid or the control plasmid and treated them with or without PA for 24 hours.

Epidemiological studies have shown that long-term HFD consumption is an important risk factor for T2DM [36], which led us to wonder whether the dose and/or duration of PA treatment would affect the protein expression of HBP1. We treated L02 and AML12 cell lines with various concentrations (0, 25, 50, 100, or 200 nm) of PA for 24 hours, and found that as the dose of PA was increased, the protein level of HBP1 gradually decreased (Figure 5G). Following this, we determined the effects of treatment with 50 nm PA for 0, 6, 12, 18, or 24 hours on HBP1 protein expression in the L02 and AML12 cell lines. As shown in Figure 5H, the protein level of HBP1 increased during the first 12 hours, but significantly decreased subsequently. These results suggest that the long-term consumption of a large amount of PA would significantly reduce the protein expression of HBP1 and the transcriptional activation of the IGFBP1 promoter by HBP1.

### The HBP1-IGFBP1 axis increases extracellular glucose concentration by reducing activation of the PI3K-AKT signaling pathway

**Figure 6:**
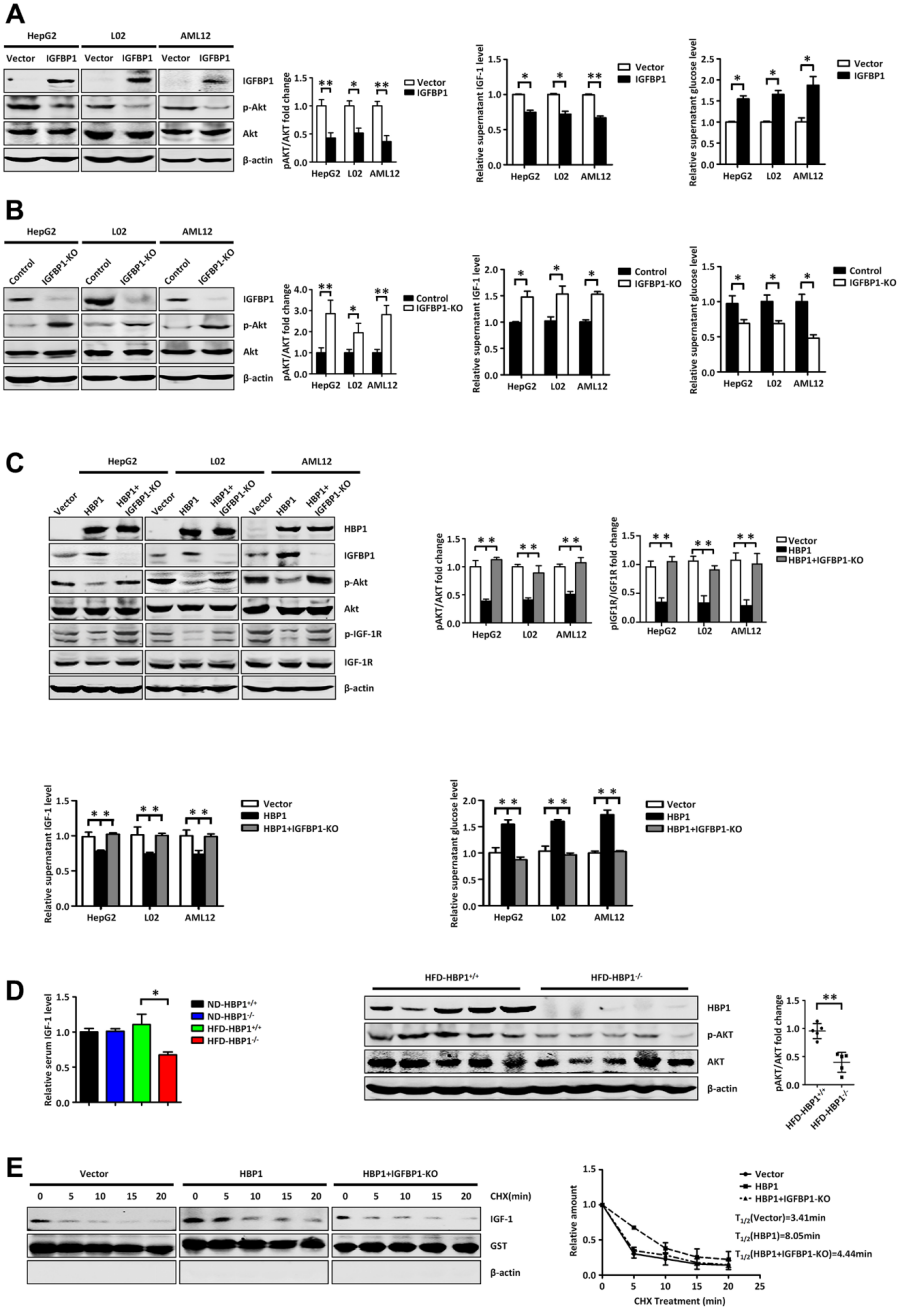
**The HBP1-IGFBP1 axis increases extracellular glucose concentration by reducing activation of the PI3K-AKT signaling pathway.** (**A**) IGFBP1 inhibits p-AKT signaling pathway, inhibits supernatant IGF-1 levels and elevates supernatant glucose level. The protein levels of IGFBP1, p-AKT and AKT were measured by western blotting in HepG2, L02 and AML12 cells. β-actin was used as a loading control. The content of IGF-1 in supernatant was measured by ELISA kit. The supernatant was changed from DMEM to EBSS and was collected after 24 hours. Then the supernatant glucose level was measured by glucose testing kit. (**B**) *IGFBP1* knockout activates p-AKT signaling pathway, increases supernatant IGF-1 levels and suppresses supernatant glucose level. The protein levels of IGFBP1, p-AKT and AKT were measured by western blotting in *IGFBP1*-knockout HepG2, L02 and AML12 cells constructed by CRISPR/Cas 9 system. β-actin was used as a loading control. The content of IGF-1 in supernatant was measured by ELISA kit. The supernatant was changed from DMEM to EBSS and was collected after 24 hours. Then the supernatant glucose level was measured by glucose testing kit. (**C**) HBP1 can inhibit p-AKT signaling pathway, decreases supernatant IGF-1 levels and elevates supernatant glucose level by IGFBP1. The protein levels of HBP1, IGFBP1, p-AKT, AKT, p- IGF-1R and IGF-1R were measured by western blotting in HepG2, L02 and AML12 cells. β-actin was used as a loading control. The content of IGF-1 in supernatant was measured by ELISA kit. Then the supernatant was changed from DMEM to EBSS and was collected after 24 hours. The supernatant glucose level was measured by glucose testing kit. (**D**) The level of serum free IGF-1 in HFD-HBP1^−/−^ mice is lower than that of HFD-HBP1^+/+^ mice (n=5), so that the PI3K-AKT signaling pathway is inhibits. The content of mice serum IGF-1 was measured by ELISA kit. The protein levels of HBP1, p-AKT and AKT were measured by western blotting. β-actin was used as a loading control. (**E**) HBP1 affects the stability of IGF-1 through IGFBP1. The protein levels of IGF-1, GST and β-actin were measured by western blotting. GST was used as a loading control. Data were the mean ± SD by a two-tail, unpaired Student’s t-test. *, p<0.05. **, p<0.01.

To further explore the roles of HBP1 and IGFBP1 in the regulation of glucose metabolism, we first aimed to characterize the relationship between IGFBP1 and extracellular glucose concentration. We overexpressed IGFBP1 in HepG2, L02, and AML12 cells, and found that the phosphorylation of AKT was lower (Figure 6A, left panel), suggesting lower activation of the PI3K-AKT signaling pathway. We next measured the concentration of free IGF-1 in the medium by ELISA, and found that the overexpression of IGFBP1 reduced the free IGF-1 concentration (Figure 6A, middle panel). Then, 24 hours after the transfection of the IGFBP1-expressing plasmid, we replaced the DMEM medium with EBSS medium, and 24 hours later collected the medium and measured its glucose concentration. As shown in the right panel of Figure 6A, IGFBP1 overexpression increased the extracellular glucose concentration. Therefore, we propose that IGFBP1 overexpression reduces the concentration of free IGF-1 in the medium and the activation of the downstream PI3K-AKT signaling pathway, causing an inhibition of glucose uptake from the medium, leading to an increase in its glucose concentration.

To further test our hypothesis, we knocked out IGFBP1 in HepG2, L02, and AML12 cells using the CRISPR/Cas9 system: we used an sgRNA targeting the second exon of the *IGFBP1* gene to reduce its transcription. As shown in Figure 6B, knockout of IGFBP1 increased the activation of the PI3K-AKT signaling pathway and the free IGF-1 concentration in the medium, and reduced the glucose concentration in the medium. Thus, IGFBP1 may increase extracellular glucose concentration by reducing the free IGF-1 concentration, leading to a reduction in the activation of the PI3K-AKT signaling pathway.

Because HBP1 increases the expression of IGFBP1, we next wondered whether HBP1 would affect the extracellular glucose concentration through its effect on *IGFBP1* transcription. Therefore, we transfected the HBP1-expression plasmid or the control vector into wild-type or IGFBP1-deficient HepG2, L02, and AML12 cells. As shown in Figure 6C, HBP1 overexpression reduced the phosphorylation of AKT and IGF-1R, and simultaneous knockout of IGFBP1 prevented this reduction (top panel). In addition, the overexpression of HBP1 reduced the free IGF-1 concentration in the medium, but simultaneous knockout of IGFBP1 prevented this reduction (bottom, left panel). Furthermore, HBP1 overexpression increased the extracellular glucose concentration, but there was no effect of simultaneous IGFBP1 knockout (bottom, right panel). These findings imply that HBP1 increases the extracellular glucose concentration by increasing the expression of IGFBP1, which inhibits activation of the IGF-1-PI3K-AKT signaling pathway.

We next wondered whether this regulation of the extracellular glucose concentration by HBP1 could be replicated *in vivo*. Therefore, we measured the free IGF-1 concentration in serum samples collected from the groups of mice described above (Figure 2) by ELISA. The left panel of Figure 6D shows that the serum free IGF-1 concentration in HFD-HBP1^−/−^ mice was lower than that of HFD-HBP1^+/+^ mice, but there was no difference between the ND-HBP1^−/−^ and ND-HBP1^+/+^ mice. We next measured the expression of key proteins in the livers of HFD-HBP1^−/−^ mice and HFD-HBP1^+/+^ mice by western blotting, and found that the PI3K-AKT signaling pathway was significantly inhibited in HFD-HBP1^−/−^ mice (Figure 6D, right panel). As shown above, the diabetes of the HFD-HBP1^−/−^ mice was significantly worse than that of HFD-HBP1^+/+^ mice. When taken together with the results shown in Figure 6D, we considered that this difference may be explained by a low serum free IGF-1 concentration, which leads to lower activation of the PI3K-AKT pathway.

Next, we aimed to determine the reason for the low serum concentration of free IGF-1 in HFD-HBP1^−/−^ mice using wild-type AML12 cells, AML12 cell lines stably expressing HBP1, and IGFBP1-knockout AML12 cell lines stably expressing HBP1. We treated the cells with cycloheximide for up to 2 h, then collected the medium and analyzed the IGF-1 concentration using western blotting. As shown in Figure 6E, the half-life of IGF-1 in control, HBP1 overexpression and HBP1 overexpression+IGFBP1 knockout cells was 3.41, 8.05 and 4.44 minutes, respectively. We also performed ELISA to test IGF-1 half-life in medium (Supplementary Figure 4). HBP1 increased IGF-1 half-life, and IGFBP1 knockdown reversed this effect. The data indicate that HBP1 overexpression increased the stability of the IGF-1 in the medium, but this increase was less marked in IGFBP1-deficient cells. This implies that HBP1 increases the stability of IGF-1 in the medium through an effect on IGFBP1. As shown in Figure 6C, we found that HBP1 overexpression reduces the concentration of free IGF-1 in the medium. Previous studies have shown that IGFBP1 binds to IGF-1, thereby preventing it from activating the IGF-1 receptor (IGF-1R) when in the free state, but this also prolongs the half-life of the IGF-1 [20, 28]. Therefore, we inferred that HBP1 reduces the free IGF-1 concentration but increases the stability of the IGF-1 in the medium through its effect on IGFBP1 expression. In addition, Figure 6E shows that IGF-1 has a short half-life. Therefore, we speculate that the free IGF-1 in HFD-HBP1^−/−^ mice may have a shorter half-life, because of the loss of the protective effect of HBP1 on IGF-1 through its effect on *IGFBP1*, resulting in lower stability of IGF-1 and the degradation of much of the excess free IGF-1.

## DISCUSSION

**Figure 7:**
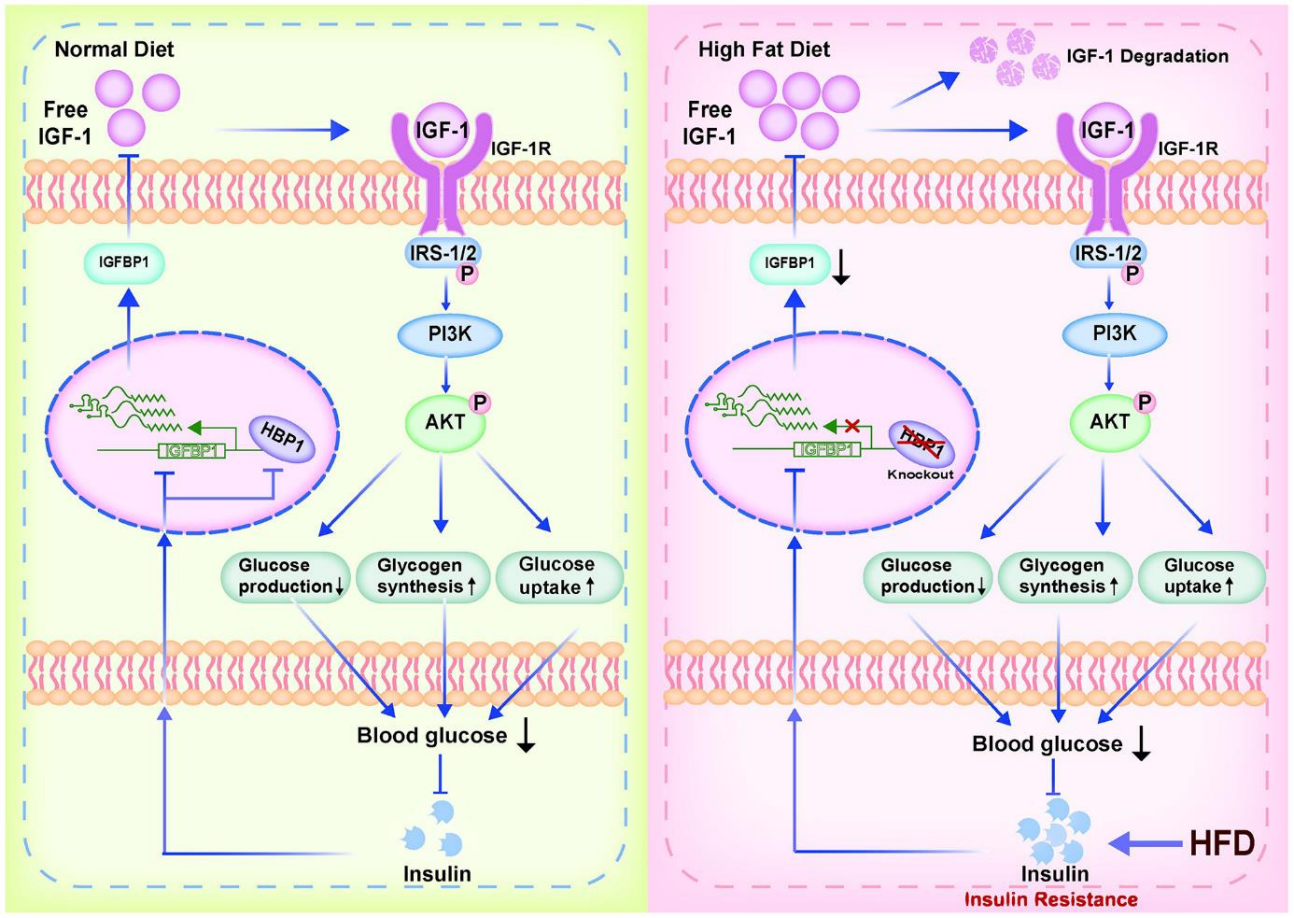
**Model of the effect of the transcription factor HBP1 on glucose metabolism and the development of HFD-induced T2DM.** Under normal physiological conditions, knockout of the HBP1 gene only causes a temporary decrease in blood glucose level, but then the insulin/HBP1/IGFBP1/IGF1/PI3K/AKT negative feedback loop ensures that the blood insulin concentration quickly recovers. However, during long-term HFD-feeding, the hypoglycemic effect of IGF-1 impairs. This would result in hyperglycemia and further stimulate insulin secretion, creating a vicious cycle, leading to insulin resistance and the development of T2DM.

In the present study, we have investigated the effect of the transcription factor HBP1 on glucose metabolism and the development of HFD-induced T2DM, and the mechanism involved (Figure 7). We found that the HBP1 protein expression is low in the livers of mice with T2DM. Although HBP1 does not affect the glucose metabolism of normal mice, HFD-fed HBP1 knockout mice show a worse diabetic phenotype than HFD-fed wild-type mice. These findings imply that HBP1 participates in glucose metabolism and affects the development of T2DM.

A large number of previous studies [20, 27] have shown that IGFBP1 is released into the circulation after being synthesized in the liver. Furthermore, the affinity between IGFBP1 and IGF-1 in the circulation is much higher than that between IGF-1 and IGF-1R. Therefore, an increase in the concentration of IGFBP1 would reduce the binding between IGF-1 and IGF-1R and inhibit downstream signaling. In addition, IGFBP1 maintains the stability of IGF-1 by binding it, thereby prolonging its half-life, delaying its degradation, and permitting its transport to target organs. Thus, IGFBP1 protects, stores, and transports IGF-1. In this way, IGFBP1 can regulate the circulating free IGF-1 concentration according to the body’s needs, enabling the circulating IGF-1 concentration to fluctuate slightly in response to external stimuli [20]. The IGF-1/IGF-1R/insulin receptor substrate (IRS) signaling pathway and the insulin/insulin receptor/IRS pathway both regulate glucose metabolism [21, 22] through PI3K and AKT, promote glycogen synthesis in liver cells, inhibit glucose production, promote the uptake of glucose from the blood, and reduce blood glucose [26, 27, 38].

Under normal physiological conditions, insulin is the key regulator of blood glucose concentration. Many studies have shown that insulin can inhibit the expression of *IGFBP1* through the IRE in the *IGFBP1* promoter region [31]. The present findings show that HBP1 activates the transcription of the *IGFBP1* gene and increases the amount of IGFBP1 protein synthesized by hepatocytes. In addition, we found that insulin not only inhibits the expression of HBP1, but also the binding of HBP1 to the *IGFBP1* promoter. Thus, insulin can inhibit the expression of IGFBP1 in more than one way, causing a reduction in the circulating IGFBP1 concentration.

IGFBP1 binds free IGF-1, thereby reducing the free IGF-1 concentration and reducing the binding of IGF-1 to IGF-1R, causing lower activation of the PI3K-AKT signaling pathway. However, the inhibitory effects of insulin on IGFBP1 expression and its direct effect to activate the PI3K-AKT signaling pathway both cause a reduction in blood glucose concentration. Such a decrease in blood glucose concentration causes pancreatic β cells to secrete less insulin [39, 40], and thus an insulin/HBP1/IGFBP1/IGF-1/PI3K/AKT negative feedback regulation loop is created. Under normal physiological conditions, knockout of the HBP1 gene only causes a temporary decrease in blood glucose concentration, which is followed by a decrease in insulin secretion, but the negative feedback loop ensures that the blood insulin concentration quickly recovers. Therefore, the deletion of the *HBP1* gene in mice does not affect their glucose metabolism under normal circumstances.

Obesity is one of the most important risk factors for T2DM, and HFD-feeding is one of the common experimental methods of inducing a T2DM-like condition in mice [36, 41]. Long-term HFD-feeding causes a substantial increase in insulin secretion in mice [3, 40], which has adverse effects on the regulation of glucose metabolism. Long-term hyperinsulinemia and HFD-feeding induces insulin resistance in the liver and other organs and increases blood glucose concentration, which can eventually progress to frank diabetes [36, 41]. According to the proposed negative feedback loop model, this marked increase in insulin secretion would inhibit the expression of the *IGFBP1* gene, thereby reducing the circulating IGFBP1 concentration and causing an increase in the concentration of free IGF-1. This increase would cause further activation of the PI3K/AKT signaling pathway via IGF-1R, leading to a reduction in blood glucose concentration and an inhibition of insulin secretion in the short term. However, during long-term stimulation in the form of HFD-feeding, the regulation of insulin secretion via this negative feedback loop could not have marked effects.

Over the long term, excessive food consumption is known to stimulate insulin release, resulting in chronic hyperinsulinemia [42]. This would significantly inhibit the synthesis of IGFBP1 protein through a variety of mechanisms, resulting in a marked decrease in the secretion of IGFBP1 protein and a reduction in the storage of IGF-1 by IGFBP1. Owing to the short half-life of IGF-1, a large amount of the hormone that would otherwise be bound to IGFBP1 would become liable to degradation. Therefore, the hypoglycemic effect of IGF-1 would be impaired in response to long-term HFD-feeding. The hyperglycemia would further stimulate insulin secretion, creating a vicious cycle, leading to insulin resistance and the development of T2DM.

In addition, HFD consumption causes an increase in the PA content of the body [36]. In the present study, we found that PA reduces the expression of HBP1 protein in the same way as insulin, by inhibiting the binding of HBP1 to the IGFBP1 promoter, thereby inhibiting the transcriptional activation of *IGFBP1* by HBP1. Therefore, HFD-feeding may also reduce the expression of IGFBP1 by increasing the concentration of PA, further predisposing toward T2DM.

During the induction of T2DM by HFD-feeding, HBP1 knockout mice were unable to fully activate transcription from the *IGFBP1* gene, resulting in significantly lower synthesis of IGFBP1 than in wild-type mice. This would make the negative feedback regulatory loop collapse early in HBP1 knockout mice when challenged by an HFD. This would explain the earlier loss of the hypoglycemic effect of IGF-1, earlier disruption of glucose homeostasis, and earlier development of T2DM identified in the HBP1 knockout mice.

In the present study, we found that the expression of HBP1 protein is low in the livers of diabetic mice. Furthermore, we have provided evidence that the negative feedback regulatory loop comprising insulin/HBP1/IGFBP1/IGF-1/PI3K/AKT plays a role in the development of T2DM. We have also shown that HBP1 is regulated by both insulin and PA. We propose that small molecules should be identified that can increase HBP1 protein expression or promote the transcriptional activation of IGFBP1, on the basis of their molecular structure and activities. It may also be possible to design personalized preventive, diagnostic, and treatment modalities for patients with T2DM or those who are predisposed toward T2DM who have low *HBP1* gene expression. In the future, it may also be possible to measure the expression of the *HBP1* gene as part of an evaluation of the risk of T2DM to prevent the development of this disease.

In conclusion, we have elucidated the mechanism whereby HBP1 participates in a negative feedback regulatory loop that affects the development of T2DM. The present findings expand our understanding of the molecular mechanisms of T2DM and provide new concepts regarding T2DM and potential drug targets for its treatment. In addition, the data suggest that HBP1 may play a role in the development of other diseases in addition to tumors, such as diabetes.

## MATERIALS AND METHODS

### Cell culture, transfection, lentivirus infection and CRISPR/Cas 9

HEK293T, HepG2, PLC/PRF/5, L02 and AML12 cell lines were cultured at 37° C, 5% CO_2_ in DMEM with 10% fetal bovine serum (FBS). Transiently transfection and lentivirus plasmid pLL3.7-shHBP1 construction were performed as described previously [8]. The sgRNA (5’-CAGTACCTATGATGGCTCGA-3’) of IGFBP1 was designed by Zhang Feng library and was inserted in CRISPR vector px459-puro, which can be screened by puromycin after transfection.

### Animals

The animal strain and breeding conditions was performed as described previously [8]. To induce T2DM, 6 weeks old HBP1-knockout male mice and wild-type (WT) littermates were fed by high fat diet (fat energy ratio 45%) for at least 3 months.

### Glucose, insulin and pyruvate tolerance tests

Oral glucose tolerance test (OGTT) was performed when mice were fasted for 6 h and glucose (5 mg/kg body weight) was infused by gavage with a feeding tube. Insulin tolerance test (ITT) was conducted when mice were fasted for 4 h, and insulin (1 U/kg body weight) was administered intraperitoneally. As for the pyruvate tolerance test (PTT), mice were fasted for 16 h, and sodium pyruvate (0.75 g/kg body weight) was administered intraperitoneally. Plasma glucose level was measured from tail vein by Roche blood glucose meter and was monitored every thirty minutes within two hours.

### Liver analysis and staining

We used freezing microtome sections of mouse liver for Oil Red O staining and paraffin sections of mouse liver for Hematoxylin and eosin (H&E) and Immunohistochemistry staining. The sections for immunohistochemistry were incubated with anti-HBP1 specific antibodies, followed by biotin labeled secondary antibodies and colored by streptavidin-ALP system.

### Western blotting and antibodies

The western blotting assay was performed as described previously [8]. The antibodies we used were as follow: HBP1 (11746-1-AP, Proteintech), IGF-1 (sc-518040, Santa Cruz Biotechnology), IGFBP1 (sc-55474, Santa Cruz Biotechnology), AKT (#9272, Cell Signaling), p-AKT (#4060, Cell Signaling), GSK-3α/β (#5676, Cell Signaling), p-GSK-3α/β (#9331, Cell Signaling), FLAG (F1804, Sigma-Aldrich), HA (MMS101P, Covance), β-actin (AC026, Abclonal), anti-mouse IgG antibody DyLight 800 (610-145-121, Rockland) and anti-rabbit IgG antibody DyLight 800 (611-145-002, Rockland).

### Real-time PCR (RT-PCR)

Total RNA was isolated from mouse liver tissues, human HepG2, PLC/PRF/5, L02 cells and murine AML12 cells. The real-time PCR assay was performed as described previously [8]. The sequence of qPCR primers that we used were as follow: human HBP1, 5’-TGAAGGCTGTGATAATGAGGAAGAT-3’ and 5’-CATAGAAAGGGTGGTCCAGCTTA-3’; human IGFBP1, 5’-TTGGGACGCCATCAGTACCTA-3’ and 5’-TTGGCTAAACTCTCTACGACTCT-3’; human β-actin, 5'-GGATGCAGAAGGAGATTACTGC-3’ and 5'-CCACCGATCCACACAGAGTA-3'; mouse HBP1, 5’-AGTTGCTGCAGTGTAATGAGAATTG-3’ and 5’-GGTGAGTATTTTCCGGTATATCTGAGG-3’; mouse IGFBP1, 5’-CTGCCAAACTGCAACAAGAATG-3’ and 5’-GGTCCCCTCTAGTCTCCAGA-3’; mouse GcK, 5'-TATGAAGACCGCCAATGTGA-3’ and 5'-CACTGAGCTCTCATCCACCA-3'; mouse Pck2, 5’-ATCTTTGGTGGCCGTAGACCT-3’ and 5’-CCGAAGTTGTAGCCGAAGAA-3’; mouse IRS1, 5’-CTCTACACCCGAGACGAACAC-3’ and 5’-TGGGCCTTTGCCCGATTATG-3’; mouse IRS2, 5’-CTGCGTCCTCTCCCAAAGTG-3’ and 5’-GGGGTCATGGGCATGTAGC-3’; mouse SREBF1, 5’-GGAGGCAGAGAGCAGAGATG-3’ and 5’-TTGCGATGTCTCCAGAAGTG-3’; mouse Fasn, 5’-CTGCCACAACTCTGAGGACA-3’ and 5’-CGGATCACCTTCTTGAGAGAC-3’; mouse PPARγ, 5’-CTGGCCTCCCTGATGAATAA-3’ and 5’-CGCAGGTTTTTGAGGAACTC-3’; mouse CPT1α, 5’-ATCGTGGTGGTGGGTGTGATAT-3’ and 5’-ACGCCACTCACGATGTTCTTC-3’; mouse Acadm, 5’-GGAGTACCCGTTCCCTCTCAT-3’ and 5’-AGGCATTTGCCCCAAAGA-3’; mouse G6pc, 5’-TTACCAAGACTCCCAGGACTG-3’ and 5’-GAGCTGTTGCTGTAGTAGTCG-3’; mouse Acaca, 5’-GCCTCTTCCTGACAAACGAG-3’ and 5’-TGACTGCCGAAACATCTCTG-3’; mouse Pdk4, 5’-GCATTTCTACTCGGATGCTCATG-3’ and 5’-CCAATGTGGCTTGGGTTTCC-3’; mouse β-actin, 5’-AGCCATGTACGTAGCCATCC-3’ and 5’-GCTGTGGTGGTGAAGCTGTA-3’. β-actin was applied as an internal control to normalized the other mRNA expression.

### Reporter gene assay and chromatin immunoprecipitation (ChIP)

The reporter gene assay and the ChIP assay were performed as described previously [8]. The primer sequences for IGFBP1 promoter were 5’- CTTTAACTGAGGGCCTGAACCCCC -3’ and 5’- CTGGACACAGCGCGCACCTTATAAAG -3’.

### Statistical analysis

SPSS software was used for statistical analysis. The data were analyzed as mean ± S.D. from at least three independent experiments. Stained areas were quantitated by Image J software. The two-tailed, Student’s t-test was used to compare the differences between two groups. One-way ANOVA was performed to the comparisons among more than two groups. P value < 0.05 was considered to be statistically significant. *, p < 0.05. **, p < 0.01.

## Supplementary Material

Supplementary Figures
