# Supplementary material for: HBP1 inhibits the development of type 2 diabetes mellitus through transcriptional activation of the IGFBP1 gene
Source: Aging (Albany NY). 2022 Nov 2;14(21):8763–82. doi: 10.18632/aging.204364 (PMC9699764; doi:10.18632/aging.204364)
Supplement: Supplementary Figures [file aging-14-204364-s001.pdf]

SUPPLEMENTARY FIGURES

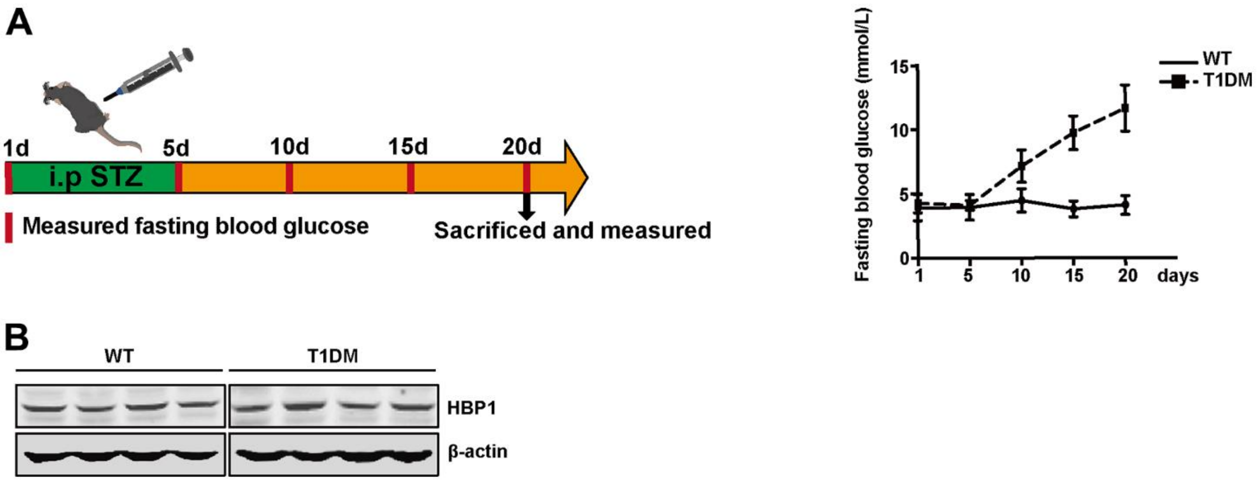

Supplementary Figure 1. Hepatic HBP1 expression remains unchanged in T1DM mouse model. (**A**) Mouse models of T1DM were induced through enterocoelia limosis injection of streptozotocin. 8 male C57BL /6J mice were randomly divided into two groups, including WT group and T1MD group. T1MD group mice received intraperitoneal injection continuously for 5 days with STZ solution (50 mg/kg), WT group mice received intraperitoneal injection with citric acid buffer. Fasting blood-glucose of the mice were measured subsequently on the 1st, 5th, 10th, 15th and 20th days. (**B**) HBP1 protein level is unchanged in T1MD mice. The protein of endogenous HBP1 was extracted from WT group and T1MD group mice (n=4) livers and was measured by western blotting. β-actin was detected as a loading control.

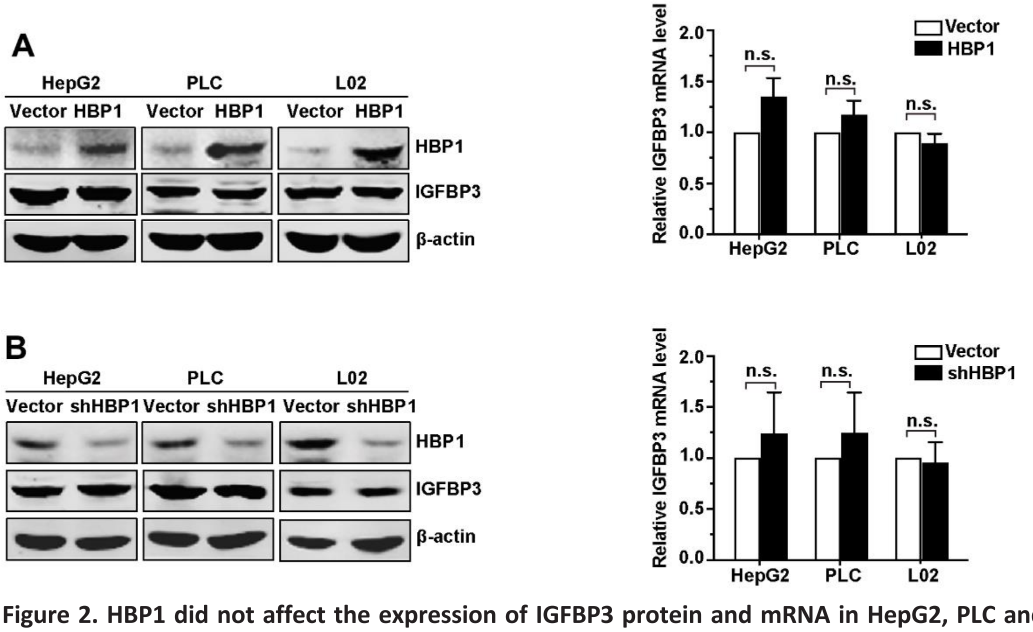

Supplementary Figure 2. HBP1 did not affect the expression of IGFBP3 protein and mRNA in HepG2, PLC and L02 cells. (**A**) Overexpression of HBP1 does not promote the expression of IGFBP3 protein and mRNA in HepG2, PLC and L02 cells. The cells were transfected with HA-HBP1 or pcDNA3 (as a control). The protein levels of HBP1 and IGFBP3 were measured by western blotting (left panel). β-actin was used as a loading control. Quantification was normalized to β-actin. The mRNA level of IGFBP3 was measured by real-time PCR (right panel). (**B**) HBP1 knockdown is not inhibit both protein and mRNA expression of IGFBP3 in HepG2, PLC and L02 cells. The cells were stably transfected with pLL3.7-shHBP1 or pLL3.7 (as a control) through lentiviral infection. The protein levels of HBP1 and IGFBP3 were measured by western blotting (left panel). β-actin was used as a loading control. Quantification was normalized to β-actin. The mRNA level of IGFBP3 was measured by real-time PCR (right panel). Data were the mean ± SD by a two-tail, unpaired Student's t-test. *, $p<0.05$. **, $p<0.01$.

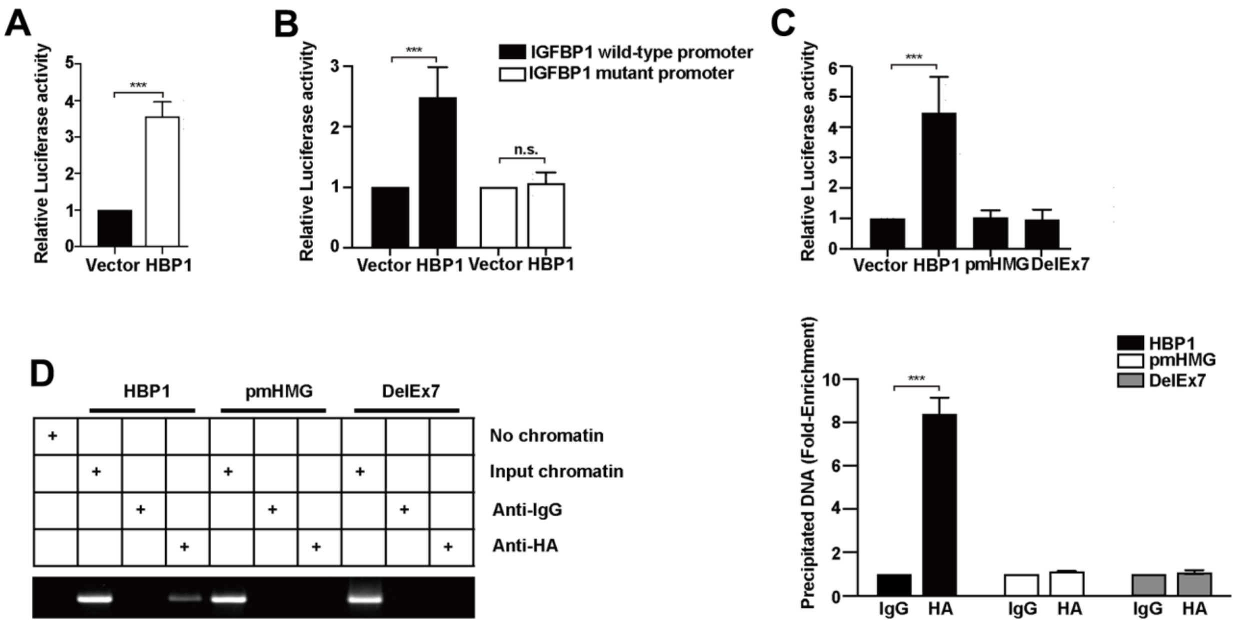

Supplementary Figure 3. HBP1 activates the IGFBP1 gene through binding an affinity site in the IGFBP1 promoter. (**A**) The relative luciferase activities of HBP1 on *IGFBP1* promoter. HepG2 cells were co-transfected with the *IGFBP1* promoter and HA-HBP1 plasmid. Luciferase activities were determined 24 hours after transfection and were analyzed from four separate experiments. (**B**) The relative luciferase activities of HBP1 on wild-type and mutant IGFBP1 promoter were analyzed in HepG2 cells. (**C**) Wild-type HBP1 rather than its mutants activates IGFBP1 promoter activities. HepG2 cells were co-transfected with wild type IGFBP1 promoter and HBP1 or its mutant plasmids. The luciferase activities were analyzed from four separate experiments. (**D**) HBP1 binding to the endogenous IGFBP1 promoter requires the HMG domain. ChIP assays were used to test the binding of exogenous HBP1 to endogenous IGFBP1 gene. HepG2 cells were transfected with HA-HBP1, HA-pmHMG or HA-DelEx7. The region from position -224 to -6 contains the HBP1 affinity site and was analyzed by specific PCR (left panel) and Realtime-PCR (right panel). Error bars represent S.D. *, $p < 0.05$, **, $p < 0.01$, ***, $p < 0.001$.

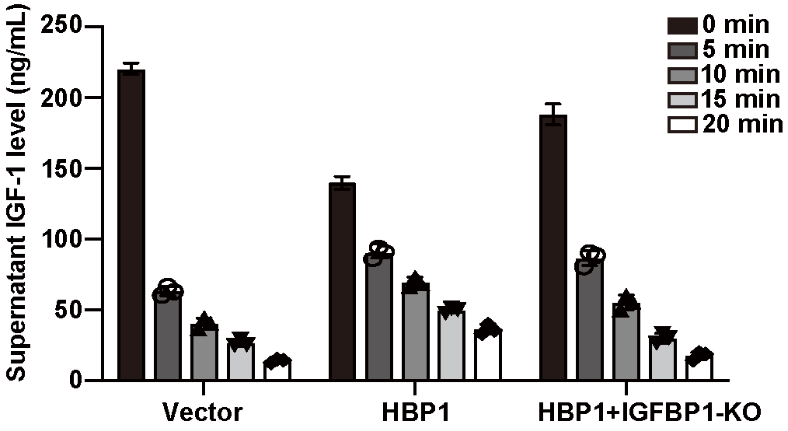

Supplementary Figure 4. HBP1 affects the stability of serum free IGF-1 through IGFBP1. Indicated HepG2 cells were treated with the cycloheximide (CHX) for 0, 5, 10, 15 and 20 min separately at final concentration of 100 μg/ml. Then The supernatant was changed from DMEM to EBSS and was collected after 24 hours. The content of IGF-1 in supernatant was measured by ELISA kit.
